# Feeder Cell Type Affects the Growth of In Vitro Cultured Bovine Trophoblast Cells

**DOI:** 10.1155/2017/1061589

**Published:** 2017-05-24

**Authors:** Islam M. Saadeldin, Ahmed Abdelfattah-Hassan, Ayman Abdel-Aziz Swelum

**Affiliations:** ^1^Department of Animal Production, College of Food and Agricultural Sciences, King Saud University, Riyadh 11451, Saudi Arabia; ^2^Department of Physiology, Faculty of Veterinary Medicine, Zagazig University, Zagazig 44519, Egypt; ^3^Department of Anatomy and Embryology, Faculty of Veterinary Medicine, Zagazig University, Zagazig 44519, Egypt; ^4^Department of Theriogenology, Faculty of Veterinary Medicine, Zagazig University, Zagazig 44519, Egypt

## Abstract

Trophectoderm cells are the foremost embryonic cells to differentiate with prospective stem-cell properties. In the current study, we aimed at improving the current approach for trophoblast culture by using granulosa cells as feeders. Porcine granulosa cells (PGCs) compared to the conventional mouse embryonic fibroblasts (MEFs) were used to grow trophectoderm cells from hatched bovine blastocysts. Isolated trophectoderm cells were monitored and displayed characteristic epithelial/cuboidal morphology. The isolated trophectoderm cells expressed mRNA of homeobox protein* (CDX2)*, cytokeratin-8* (KRT8)*, and interferon tau* (IFNT)*. The expression level was higher on PGCs compared to MEFs throughout the study. In addition, primary trophectoderm cell colonies grew faster on PGCs, with a doubling time of approximately 48 hrs, compared to MEFs. PGCs feeders produced a fair amount of 17*β*-estradiol and progesterone. We speculated that the supplementation of sex steroids and still-unknown factors during the trophoblasts coculture on PGCs have helped to have better trophectoderm cell's growth than on MEFs. This is the first time to use PGCs as feeders to culture trophectoderm cells and it proved superior to MEFs. We propose PGCs as alternative feeders for long-term culture of bovine trophectoderm cells. This model will potentially benefit studies on the early trophoblast and embryonic development in bovines.

## 1. Introduction

There are two distinct cell populations in the blastocysts of mammals, the trophoblast and the inner cell mass. During embryogenesis, trophoblast cells are believed to be the first to differentiate [1]. The trophoblastic cells form most of the placenta, whereas the inner cell mass grows to give the embryo and its related membranes [2]. So, although the implantation and placental formation step is dependent on the differentiation of the trophoblast, this process involves unknown participating factors and remains weakly understood. Recently, a number of specific molecules has been linked as markers to trophoblastic cells, during bovine peri-implantation processes, such as interferon tau* (IFNT)* [3, 4], early trophoblastic marker* CDX2* [5], and cytokeratin-8* (KRT8)* in in vitro produced bovine embryos [6].

Studying development and functions of trophoblastic cells is critical; therefore, in vitro models for culturing primary trophoblast cells are essential. However, the finite lifespan of primary trophoblast cells limits their long-term culture and use in investigations. In an attempt to prolong trophoblast cells lifespan in goat, telomerase immortalized trophoblast cells (hTERT-transfected cells) showed more telomerase activity and proliferated persistently for at least 50 passages without any signs of senescence [7]. But, many researchers are skeptic about using transfected cell lines in their research because their regeneration is costly and complex, and little is known about how transfection could inadvertently change the outcomes of their research. Consequently, maintenance of trophoblastic cell lines using feeder cells comes as a more convenient and feasible approach. In this regard, recently, some trophoblastic cell lines were established, on mouse embryonic fibroblasts as feeders, in several species as porcine [8], murine [9], and bovine [10, 11]. Henceforward, improving the culture system is of particular interest and advantage, to facilitate the in vitro investigation of the trophoblastic cells. In the present study, and for the first time, porcine granulosa cells were used as alternative feeder cell for culturing primary trophoblastic cells which were isolated from in vitro produced bovine blastocysts.

## 2. Materials and Methods

### 2.1. Oocyte Collection and In Vitro Maturation (IVM)

Cow ovaries were obtained from a nearby abattoir, washed with saline and kept in it at 35°C, and promptly sent (within 2 hrs) to the lab. These cows' management was as previously described [12]. Ovarian follicles (diameter = 2–8 mm) were aspirated with an 18-gauge needle connected to 10 mL disposable syringe to obtain cumulus-oocyte complexes (COCs). The COCs were selected, if they had evenly granulated cytoplasm and enclosed by three or more layers of compact cumulus cells and washed 3x in HEPES-buffered TCM-199 (Invitrogen, USA) supplemented with 10% FBS, 2 mM NaHCO_3_ (Sigma, USA), and 1% penicillin-streptomycin (v/v, Sigma, USA). Followed by IVM, COCs were cultivated in 4-well plates (30–40 oocytes per well; Falcon, BD, UK) in 450 *μ*L TCM-199 with 10% FBS, 0.005 AU/mL FSH (Antrin, Tokyo, Japan), and 1 *μ*g/mL 17*β*-estradiol (Sigma, USA) at 39°C in a humidified atmosphere of 5% CO_2_ for 24 hrs.

### 2.2. In Vitro Fertilization (IVF) and In Vitro Culture (IVC) of Embryos

This protocol followed our previously published protocol [11]. Motile spermatozoa were obtained by purification using Percoll gradient method as previously described [13]. Shortly, semen straws were thawed and spermatozoa were collected by centrifugation on a (45–90%) Percoll discontinuous gradient at 1500 rpm for 15 minutes. The 45% Percoll solution was prepared using 90% Percoll and TALP medium (1 : 1 ratio). The obtained sperm-pellet was washed (2x) with TALP medium then centrifuged at 1500 rpm for 5 minutes. Then, active and motile sperms (1-2 × 10^6^ sperm/mL) were used for inseminating the IVM oocytes (day 0) for 18 hrs in 30 *μ*L IVF/TALP medium blanketed with mineral oil at 39°C in 5% CO_2_ humidified atmosphere. Plausible zygotes were stripped and cultivated in a two-step defined culture medium as previously described [14, 15] and overlaid with mineral oil (Sigma, USA). Fertilization was repeated six times (*n* = 150, 25 oocytes each). We obtained forty-nine blastocysts (32.6%); among them thirty-six blastocysts were hatched (24%) on day 10 of IVC that were used for trophoblast culture.

### 2.3. Preparation of Feeder Cells

Two different feeders were used: the conventional method using mouse embryonic fibroblasts (MEFs) as we described before [11] and new feeder porcine granulosa cells (PGCs). PGCs were obtained through aspiration of follicular fluid of 4–6 mm porcine ovarian follicles with an 18-gauge needle. The follicular fluid was centrifuged at 1500 rpm for 2 minutes and then washed three times using PBS and then with culture medium (DMEM and FBS 10%). The two cell types were mitotically inactivated using mitomycin-C (Sigma-Aldrich Corp.) followed by culturing (well density = 5 × 10^4^ cell/mL) on 4-well plates coated with 0.1% (w/v) gelatin. The medium comprised of DMEM-199 and 10% (v/v) FBS, with addition of nonessential amino acids (NEAA), *β*-mercaptoethanol, and nucleosides as previously described [10].

### 2.4. Measurement of Steroid Hormones in the Culture Medium

Steroids (estrogen and progesterone) were measured in the culture medium using commercial kits, following producer's directions. Culture media were aspirated from five different replicates and were centrifuged at 1500 rpm for 5 min. The supernatant was divided into aliquots and preserved at −20°C until analyzed. Progesterone (P4) levels were measured by radioimmunoassay (RIA) using commercial progesterone kit (Coat-a-Count, Siemens, USA). The kit contains rabbit anti-P4 antibody, and the minimum detection limit is 0.02 ng/mL. The intra- and interassay coefficient of variation (CV%) ranged from 2.7 to 8.8 and from 3.9 to 9.7, respectively. Also, estradiol (E2) levels were determined by electrochemiluminescence immune-assay with commercial kit (Estradiol II kit, Roche, USA). The kit contains rabbit anti-E2 antibody and the kit minimum detection limit is 5.0 pg/mL. The intra- and interassay coefficient of variation (CV%) ranged from 2.3 to 6.2 and from 6.2 to 13.0, respectively. Each sample from each replicate was measured three times and the data were recorded as mean ± SEM.

### 2.5. Isolation and Culture of Bovine Trophoblastic Cells

Thirty-six IVF hatched blastocysts on days 10-11 of IVC were randomly distributed into two 4-well plates (Nunc, Thermo Scientific, Denmark), grouped into MEF and PGCs groups, eighteen embryos each and three embryos per each replicate. The plates were precoated with 0.1% gelatin and followed by culturing of mitotically inactivated feeder layers of either mouse embryonic fibroblasts (MEF) or porcine granulosa cells (PGCs). The blastocysts were primary cultivated in 1 mL DMEM/F12 medium [composed of DMEM/F12 provided with 10% FBS, 0.1 mM *β*-mercaptoethanol, 1% NEAA (Invitrogen), 2 mM GlutaMax, and 1% penicillin/streptomycin (Invitrogen)], with medium changes with fresh medium every 3-4 days. Subculturing of the trophoblastic cells was by mechanical detachment and chopping of the colonies into relatively equal small pieces and recultivating those pieces on new feeder-plates. The small chops were centrifuged at 1500 rpm for 2 min and pelleted in 1.5 mL rounded bottom centrifuge tubes. Then, resuspension of the pellet was done with DMEM/F12, followed by plating onto new feeder-plates (split ratio = 1 : 4–1 : 6). Incubations were done at 39°C in 5% CO_2_ humidified atmosphere.

### 2.6. RT-PCR

Trophoblast colonies (*n* = 5, three replicates) of the 1st and 10th passages were mechanically isolated and washed three times with PBS. Total RNA was extracted from trophoblast colonies using an RNeasy total extraction kit (Qiagen, USA) following the manufacturer directions, and as we described previously [11]. Reverse transcription reactions were done in 20 *μ*l reactions at 50°C for 50 minutes using random-hexamers and SuperScript™ III Reverse-Transcriptase (Invitrogen). One *μ*g cDNA was subjected to RT-PCR using a Maxime PCR-PreMix kit (i-starTaq, Intron, Republic of Korea). Primers and their sequences, annealing temperatures and expected sizes of products are listed in Table 1. The amplification cycle was done with initial denaturation at 95°C for 5 minutes, followed by cycles of denaturation at 95°C for 30 seconds, annealing for 30 seconds, extension at 72°C for 45 seconds and final extension at 72°C for 5 minutes. PCR products (10 *μ*L) were ran on 1% agarose gel (Intron) stained with RedSafe™ (Intron). Densitometry scanning with ImageJ v1.45 software (NIH, USA) was done to quantify the intensity of RT-PCR signals and specific target's values were normalized using the internal control* (GAPDH)* to calculate relative expression units. All controls in RT-PCR, reactions without cDNA template and those without reverse transcription, gave no amplification reaction.

### 2.7. Statistical Analysis

Data were analyzed with one-way ANOVA, Tukey's test was done to conclude significant differences between the different experimental groups using GraphPad (Version 4.0). Data were considered statistically significant when *P* value was <0.05.

## 3. Results and Discussion

In this study, the primary culture of trophoblasts was done by culturing of hatching/hatched blastocysts on feeder cells, which were either mouse embryonic fibroblasts (MEFs) or, for the first time, porcine granulosa cells (PGCs).

When the feeders were cultured alone, PGCs showed higher proliferation with approximately 24 hrs doubling time comparing to MEFs (*P* ≤ 0.05). In addition, PGCs were easier to recover from monolayer cultures and offered a fair amount of sex steroids, 17*β*-estradiol (E2, 31.21 ± 3.1 ng/mL) and progesterone (P4, 6.36 ± 0.4 ng/mL). Steroids production by cultured porcine granulosa cells has been previously reported [16].

**Figure 1:**
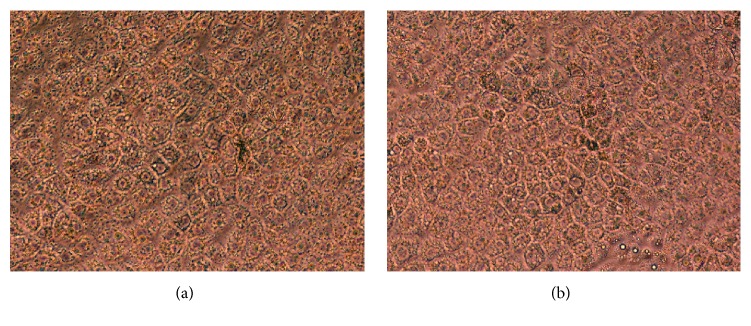
Representative phase-contrast light micrograph showing the typical cuboidal morphology of cultured bovine trophoblastic cells (10th passage) on mouse embryonic fibroblasts, MEFs (a), and on porcine granulosa cells, PGCs (b) [200x].

After coculture with mitotically inactivated MEFs or PGCs, blastocysts were seen attached and from it outgrowths were observed; then it was kept in culture for 10 days till it reached approximately 1 cm diameter. Following that, secondary and succeeding subcultures underwent mechanical detachment and cutting/chopping of these cell-growths and subculturing of the relatively similar sized small pieces/chops on fresh feeder-plates every 7 days (after reaching about 0.5 cm diameter). In all subcultures, trophoblasts were morphologically visible as large cuboidal cells under phase-contrast microscope (Figure 1).

**Figure 2:**
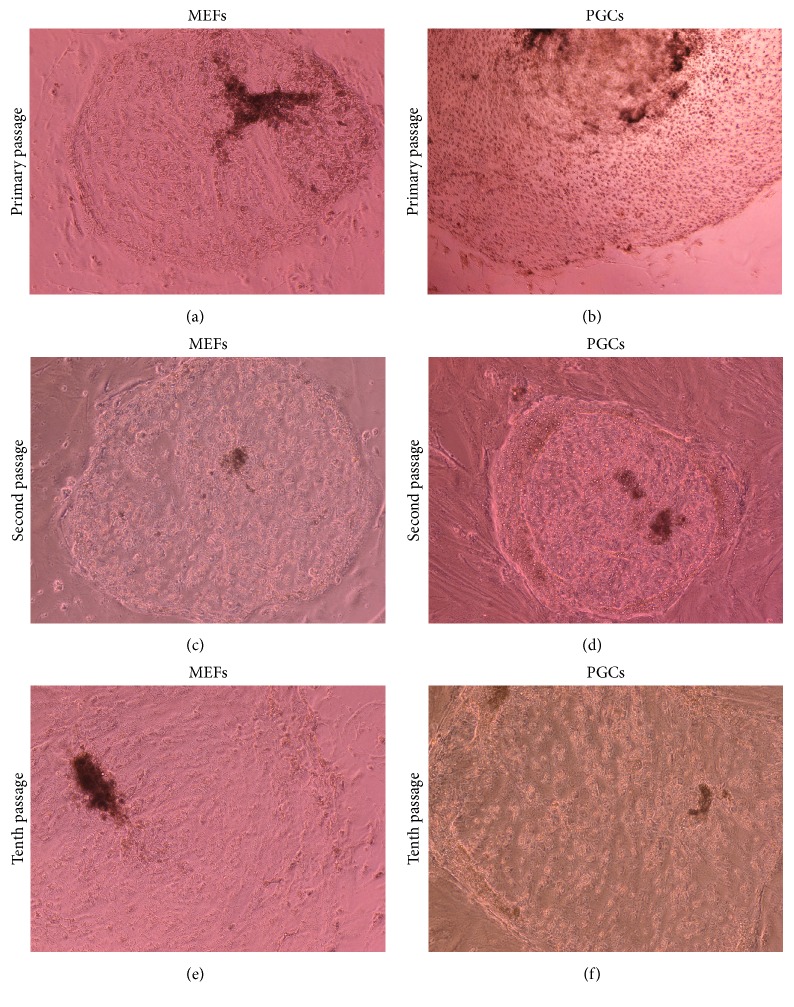
Phase-contrast light micrographs showing culture of bovine trophoblast on mouse embryonic fibroblast (MEFs) and on porcine granulosa cells (PGCs). The cells were subcultured until the 10th passage [100x].

The isolated trophoblastic cells showed faster growth pattern in primary culture on PGCs, in one-week colony diameter measured 7.0  ±  0.3 mm, compared to those cultured on MEFs; colonies grew to 5.7 ± 0.4 mm (*P* ≤ 0.05; Figures 2(a) and 2(b)), whereas, on subsequent cultures (2nd–10th) the difference in colony morphology, although it was visually larger when trophoblasts were cultured on PGCs compared to MEFs, did not reach statistical significance (*P* > 0.05; Figures 2(c), 2(d), 2(e), and 2(f)).

**Figure 3:**
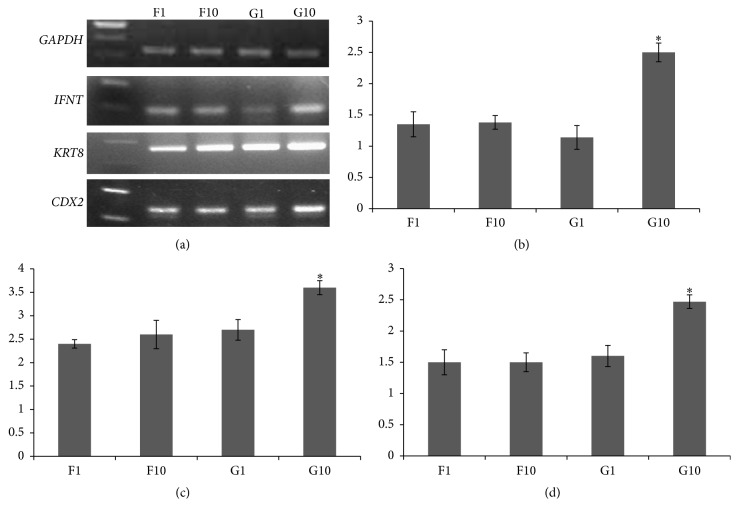
(a) RT-PCR analysis using primers specific for interferon tau* (IFNT)*, keratin-8* (KRT8),* and homeobox protein* (CDX2)* expression in trophectoderm colonies. In all analyses, reactions without cDNA template or reverse transcriptions resulted in negative amplification. ((b), (c), and (d)) Densitometric relative expression values of* IFNT*,* KRT8,* and* CDX2*, respectively (normalized to those of the internal control* GAPDH*) using ImageJ v1.45 software (NIH, USA). Trophectoderm cells were grown on mouse embryonic fibroblasts, primary culture (F1) and 10th passage (F10), and on porcine granulosa cells, primary culture (G1) and 10th passage (G10) [^*∗*^*P* value ≤ 0.05].

In contrast to the colony size, the isolated cells showed high expression of trophoblast gene-markers throughout the study; these were* IFNT*,* KRT8,* and* CDX2* (Figure 3). Of importance, the expression levels of* IFNT*,* KRT8,* and* CDX2* mRNA were higher when trophoblastic cells were cultured on PGCs compared to MEFs, particularly at the 10th passage (*P* ≤ 0.05; Figure 3). The culture was stopped at passage 10; that is, the culture duration was approximately 70 days after the primary culture.

Isolated trophoblastic cells on both feeders were morphologically similar, which visually is a good sign that culturing the cells on PGCs resulted in a normal morphology similar to using MEFS. What is more, the trophoblast cells isolated on PGCs showed superior quality, indicated by higher expression of* IFNT*,* KRT8,* and* CDX2* and long-term culture for up to 70 days. Interferon tau is produced early in preimplantation [3, 4] and its expression indicates normal trophoblast function. So, in case of PGCs as feeders, higher expression of trophoblast* IFNT* fairly indicates an improved trophoblast function, while the expression of the homeobox protein* CDX2* is considered as a healthy sign of preimplantation trophectoderm cells [5, 15] and again it was highly expressed in cells cultured on PGCs, favoring PGCs as feeders over MEFs. Moreover,* KRT8* expression as a marker for trophectoderm is also consistent with other studies on in vitro produced bovine embryos [6, 17].

In addition, PGCs provided the cultured trophoblastic cells with a considerable amount of E2 and P4. Previous work revealed that estrogens and progesterone receptors were expressed in trophoblast and caruncular epithelium and suggested that E2 and P4 are important factors controlling caruncular growth, differentiation, and function [18]. Interestingly, it was found recently that genes encoding steroid hormones were also expressed by bovine trophoblastic cells [19, 20]. Moreover, steroidogenic proteins were expressed in bovine placental tissue during the first half of gestation [21]. Hence, we assume that the constant supplementation of sex steroids and other yet to be determined factors, during trophoblasts coculture with PGCs, is better and superior to using MEFs as feeders. Nevertheless, steroids synthesis and functions during early implantation remain understudied and more research is needed to understand their roles(s) throughout the peri-implantation period.

## 4. Conclusions

In this report, we described the isolation of trophoblastic cell line from bovine IVF embryos for the first time using granulosa cells as feeders. The isolated trophoblastic cells had normal shape and superior trophoblast quality, indicated by increased expression of trophoblast markers* IFNT*,* KRT8,* and* CDX2* compared to cells isolated on MEFs. Using this model, we anticipate more studies aiming at elucidating the fetal-maternal crosstalk at implantation. Also, it will be a valuable model for studies interested in explaining effects of diseases, infections, or toxicity on the placental formation and early development and gestation.

## Figures and Tables

**Table 1:** Primers used for RT-PCR.

Gene	Primer sequences (5′-3′)	Annealing temperature (°C)	Fragment size (bp)	GenBank accession number
*IFNT*	F: TCCATGAGATGCTCCAGCAGT	60	103	X65539
	R: TGTTGGAGCCCAGTGCAGA			
*CDX2*	F: GCCACCATGTACGTGAGCTAC	60	140	DQ126146
	R: ACATGGTATCCGCCGTAGTC			
*KRT8*	F: CACCAGTTCCAAGCCTGTGG	55	176	NM_001033610.1
	R: TCAGGTCTCCTGTGCAGATGC			
*GAPDH*	F: GGCGTGAACCACGAGAAGTA	60	119	NM_001034034.1
	R: CCCTCCACGATGCCAAAGT			
